# Assessing the suitability of antibiotic resistance markers and the indirect ELISA technique for studying the competitive ability of selected Cyclopia Vent. rhizobia under glasshouse and field conditions in South Africa

**DOI:** 10.1186/1471-2180-9-142

**Published:** 2009-07-20

**Authors:** Amy C Spriggs, Felix D Dakora

**Affiliations:** 1 Botany Department, University of Cape Town, Private Bag, Rondebosch 7701, Cape Town, South Africa; 2 Chemistry Department, Tshwane University of Technology, Private Bag X680, Pretoria 0001, South Africa

## Abstract

**Background:**

Symbiotic N2 fixation in legumes is constrained by many factors, including the paucity of suitable soil rhizobia To maximise growth of legume species therefore often requires the application of effective rhizobia as inoculants. But where native strains out-compete introduced rhizobia for nodule formation, it is important that the competitiveness of selected strains is tested in the field and glasshouse prior to their recommendation as commercial inoculants. However the methodology for strain identification inside nodules has often proved difficult and thus limited this field of research. In this study, the suitability of the antibiotic resistance technique (both intrinsic low-resistance fingerprinting and high-resistance marking) and the serological indirect ELISA method were assessed for their ability to detect selected Cyclopia rhizobia under glasshouse and field conditions. The four rhizobial strains that were used, namely PPRICI3, UCT40a, UCT44b and UCT61a, were isolated from wild Cyclopia species growing in the Western Cape fynbos of South Africa.

**Results:**

The test strains formed two distinct groups with regard to their intrinsic resistance to the antibiotics streptomycin sulphate and spectinomycin dihydrochloride pentahydrate, making it impossible to use intrinsic antibiotic resistance to distinguish strains from within the same intrinsic resistance group. The use of strains marked with double antibiotic resistance was also investigated. A number of these strains lost their antibiotic marker tags after one plant passage; and some also lost their competitive ability. The indirect ELISA technique provided a more satisfactory method of identifying selected Cyclopia strains under both field and glasshouse conditions. The primary antibodies raised against strains UCT40a, UCT61a and UCT44b gave absorbance readings that were unambiguously negative (0.30 OD405), while those of strain PPRICI3 were ambiguous (0.50 OD405) with many false positive readings (1.0 A405). The indirect ELISA method showed a high level of analytical sensitivity in glasshouse experiments and there were no cross-reactions between the four test strains. The method was also suitable for detecting three of the four test strains in competition studies under field conditions, and can also be used to identify some strains under field conditions.

**Conclusion:**

The antibiotic marker method was found unsuitable for identifying Cyclopia rhizobia in competition experiments in both glasshouse and field conditions. However, the indirect ELISA technique was found suitable for identifying these strains in glasshouse studies. The method was also appropriate for identifying strains UCT40a, UCT44b and UCT61a, but not strain PPRICI3, in field competition studies.

## Background

*Cyclopia *Vent. (Fabaceae) is a shrubby perennial legume endemic to the Mediterranean heathland vegetation (fynbos) of the Western Cape of South Africa [1]. The shoots of several species of the genus have been harvested from the wild for centuries as a source of an herbal infusion known as honeybush tea. Due to its caffeine-free, flavonoid-rich, anti-oxidant properties, the demand for this tea has increased worldwide. To meet this demand requires the cultivation of *Cyclopia *as a commercial crop. Species of this genus exhibit indeterminate nodulation, and are therefore dependent on symbiotic N_2 _fixation for their N nutrition [2]. This suggests that manipulation of the symbiosis could lead to increased N nutrition, and hopefully greater tea yields in the low-nutrient environment of the Western Cape.

In Africa, symbiotic N_2 _fixation in legumes is constrained by many factors, including the paucity of suitable soil rhizobia, low concentrations of nutrients in the soil [3] and the quality of legume root exudates [4]. To maximise growth of the tea-producing *Cyclopia *species (which are adapted to highly acidic, low N and P environments) would require optimising soil conditions that enhance nodule formation and promote symbiotic N nutrition. This can be achieved via soil amelioration with exogenous nutrient inputs and/or the provision of sufficient quantities of an effective rhizobial symbiont as inoculant [5-7]. Although the initial stages of selecting high N_2_-fixing strains for inoculant production are usually conducted under controlled conditions in the glasshouse [8-10], subsequent testing is done under field conditions as biotic and abiotic factors can influence strain performance in the field, especially when in competition with indigenous native soil rhizobia. These native strains often out-compete introduced rhizobia for nodule formation in the host plant, leading to poor legume response to inoculation [11-13]. It is therefore important that the competitiveness of selected rhizobial strains is tested in the field prior to recommending their use as commercial inoculants.

The methodology of strain identification inside nodules has, however, often proved difficult, and thus limited this field of research. Three approaches that are routinely used, include 1) antibiotic resistance, 2) serological techniques, and more recently 3) genetic markers. Antibiotic resistance has traditionally been used as a marker in competition studies because the method is simple and requires no specialised equipment [14-19]. The intrinsic antibiotic resistance method can be used as a fingerprint to identify strains; just as mutants resistant to high antibiotic concentrations can be developed as markers for competition experiments.

Serological identification of rhizobial strains involves the use of antibodies raised against surface antigens of the test strain to detect the presence (or absence) of that strain in a suspension through agglutination, immunodiffusion, immunofluorescence or the enzyme-linked immunosorbent assay (ELISA). Because the antigenic properties of rhizobia are stable characteristics [24-26], the serological method is particularly useful in ecological studies as it does not modify the strain or alter its nodulation competitiveness. The immunofluorescence technique has also been successfully used to rapidly identify rhizobial strains [27-29], though this requires expensive equipment and large quantities of labelled antibody.

The ELISA technique is highly specific, reproducible, and commonly used to detect rhizobial strains directly from nodules. Additionally, the method is sensitive, can detect antigens in small nodules, uses small quantities of reagents, is relatively quick, and permits the rapid screening of large nodule samples. It can also detect double strain occupancy of nodules [30-34]. However, cross-reaction with native strains in field soils can lead to false positive results, thus limiting its application. A novel advance in strain detection has been the introduction of stable genetic markers [35-39]and DNA probes [40-43]into test rhizobial strains. However, the insertion of a foreign gene can increase the metabolic burden on the cell [44] and alter its competitive ability [45-47]. Furthermore, the release of such transgenic microbes into the environment is controversial [48-51]. The method also requires specialised knowledge and equipment and is therefore unsuitable for studies in developing countries with low-technology laboratories.

In this study, the suitability of the antibiotic resistance technique (both intrinsic low-resistance fingerprinting and high-resistance marking) and the serological indirect ELISA method were assessed for their ability to detect selected *Cyclopia *rhizobia under glasshouse and field conditions. Four rhizobial strains (PPRICI3, UCT40a, UCT44b and UCT61a) were used in this study. The strains were isolated from wild *Cyclopia *species growing in the Western Cape fynbos of South Africa. Strain PPRICI3 was isolated by the Plant Protection Research Institute (PPRI) within the Agricultural Research Council of South Africa, and is currently the recommended inoculant strain for *Cyclopia *cultivation, while strains UCT40a, UCT61a and UCT44b were isolated in our laboratory and selected for their high symbiotic performance under glasshouse conditions.

## Methods

### Bacterial strains and growth media

The rhizobia used in this study included strains UCT40a, UCT44b, UCT61a and PPRI13, which were isolated from native *Cyclopia *species in the Western Cape of South Africa, using yeast-mannitol agar as growth medium. The choice of these four strains out of 39 bacterial isolates was based on their superior symbiotic performance. In general, some of the 39 bacterial isolates were faster in growth (appearing within two days of streaking and producing copious quantities of exopolysaccharide gum, e.g. UCT44b and UCT61a), while phenotypically similar strains only appeared 5 days after streaking.

### Antibiotic Resistance

#### Intrinsic natural resistance to low antibiotic concentrations

The intrinsic resistance of the four *Cyclopia *strains to the antibiotics streptomycin sulphate (Sigma Chemical Co. Ltd.) and spectinomycin dihydrochloride pentahydrate (Fluka Biochemica Ltd.) was determined by streaking rhizobial culture onto yeast-mannitol agar (YMA^52^) plates containing incremental concentrations of streptomycin (0, 0.05, 0.1, 0.2, 0.4, 0.6, 0.8, 1.0, 1.2, 1.4, 1.6, 1.8, 2.0 and 5.0 μg ml^-1^) or spectinomycin (0, 0.2, 0.4, 0.6, 0.8, 1.0, 2.0, 5.0, 10.0 and 20.0 μg ml^-1^). The antibiotics were first sterilised by filtration through a 0.45 μm Millipore filter before addition to autoclaved YMA (cooled to < 50°C). Test strains were grown in yeast-mannitol broth (YMB^52^) at 20°C to 0.6 OD_600_, serially diluted to 10^-6 ^and 0.1 ml streaked onto each plate. Plates were streaked in triplicates. Colony-forming units (CFU) per plate were counted after four days of growth. A strain was considered to have intrinsic resistance to an antibiotic if it attained 50% or more growth on antibiotic plates (colony-forming units, CFU, per plate) compared to antibiotic-free control plates.

#### Antibiotic marking

To develop spontaneous antibiotic-resistant mutants, streptomycin or spectinomycin was incorporated at 10 × the intrinsic resistance level of the test strain into YMA plates. Unmarked parent strains were each grown in YMB to 0.6 OD_600 _and 0.1 ml (10^7 ^– 10^8 ^cells), and streaked onto five replicate streptomycin-containing YMA plates. Mutants that appeared spontaneously within five days of growth were isolated, re-streaked onto YMA containing streptomycin, and stored at 0°C. For each test strain, three streptomycin-resistant mutants were randomly selected, grown in YMB broth to 0.6 OD_600 _and 0.1 ml streaked onto each of five replicate spectinomycin-marked plates. To develop a double marker, the spontaneous mutants were isolated and re-streaked onto plates containing both antibiotics. After growth, they were re-streaked on YMA slants containing the two antibiotics and incubated to grow at 20°C for storage at 0°C. For strain PPRICI3, only streptomycin-resistant mutants were obtained, as no doubly marked colonies appeared after 10 days of growth. For strain UCT40a, only two doubly-marked colonies were obtained.

#### Integrity test using plants in Leonard jars

Leonard jar assemblies supplied with N-free 1/4 strength Hoagland's nutrient solution [53] were used to assess the competitive ability of marked strains compared to their unmarked parents. Treatments included jars inoculated with the parent strains alone, the marked strains alone and 1:1 mixtures of parent and marked strains. Uninoculated jars served as negative controls. Jars were autoclaved prior to planting with pre-germinated seedlings of *Cyclopia maculata *raised from surface-sterilized seed. C *maculata *is a fast-growing species on which all parent strains are effective. Five replicate jars were used, each with one seedling. The glasshouse provided a 12-h day and night cycle, with a temperature range of 16 – 28°C.

Treatment strains were grown in YMB to 0.6 OD_600_, diluted to 0.2 OD_600 _and each jar inoculated with 1 ml of the appropriate strain. For the mixed treatments, the strains were mixed 1:1 before inoculation. Cell numbers were estimated as CFU ml^-1 ^culture by streaking serial dilutions of the culture onto antibiotic-free YMA plates in triplicates and counting CFU after fours days of growth. Cell density across all strains ranged from 1 × 10^8 ^to 5 × 10^8 ^CFU ml^-1 ^culture.

Plants were harvested at 16 weeks and each separated into shoots, roots and nodules. Nodules were counted and weighed, while shoots and roots were oven-dried at 60°C for dry matter determination. Rhizobia were isolated from the larger nodules (5 to 10 nodules per jar) as described by Vincent^52^. Each isolate was streaked onto three replicate plates containing the appropriate concentrations of the antibiotics streptomycin and spectinomycin for the test (Table 1). Three antibiotic-free plates were included for comparison. If a nodule isolate achieved more than 50% growth on antibiotic plates relative to growth on antibiotic-free plates, it was considered resistant to the antibiotic and therefore the marked strain occupying that nodule. The number of nodules occupied by the marked strain provided a measure of its competitive ability.

**Table 1:** Levels of antibiotics used to develop resistant mutant strains of *Cyclopia*.

Antibiotic	Concentration of antibiotics used (μg.ml^-1^)			
	**PPRICI3**	**UCT40a**	**UCT44b**	**UCT61a**
Streptomycin	1	1	10	5
Spectinomycin	10	5	80	80

Nodule occupancy data were pooled for each test strain and analysed using a χ^2 ^test against a null hypothesis of 50% expected nodule occupancy for equal competitive ability between marked and parent strains. The appropriateness of data pooling was assessed using heterogeneity χ^2 ^tests [54]. For the marked strain treatments, the ability to grow on antibiotic plates provided a measure of the strains retention of antibiotic resistance after plant passage, while a reduction in growth indicated loss of the antibiotic marker.

#### Indirect ELISA technique

The indirect ELISA technique, modified from Kishinevsky and Maoz [55], was tested here for its ability to identify *Cyclopia *rhizobia under both glasshouse and field conditions. In the indirect ELISA method, the antigen is adsorbed, followed by the application of purified primary antibody and a single secondary antibody-conjugate. The antibody-conjugate (usually goat anti-rabbit conjugate) is commercially available and can be used in conjunction with a number of strain-specific antibody preparations. The method is simpler, but has lower analytical sensitivity than the direct method [55,56].

#### Production of strain-specific primary antibodies

The four test strains used in this study were grown in a defined broth medium containing 0.5 g K_2_HPO_4_, 0.2 g MgSO_4_.7H_2_0, 0.1 g NaCl, 0.5 g KHPO_4 _and 10 g mannitol in 1 l distilled water^53 ^and incubated at 20°C to obtain 0.4 OD_600_. To remove exopolysaccharides (produced in large quantities by strains UCT44b and UCT61a), the bacterial cells were washed three times by repeated centrifugation in phosphate-buffered saline (PBS) solution. The final sediment was suspended in 10 ml saline solution (150 mM NaCl) to a final concentration of > 10^9 ^CFU ml^-1^.

Antibodies were prepared against each test strain using adult New Zealand White rabbits. The rabbits were bled prior to inoculation to assess their pre-inoculation antibody levels. One rabbit was used for each test strain and was injected with the appropriate antigen according to the following protocol: Day 1: 0.5 ml intramuscular injections into each hind leg (with equal parts Freund's complete adjuvant mixed prior to injection); Day 14: 1 ml intravenous injection; Day 21: 1 ml intravenous injection; Day 28: 1 ml intravenous injection; Day 35: trial bleed to check antiserum titre; Day 37: bleed by cardiac puncture after 0.15 ml intravenous acetylpromazine (sedative) injection. Intravenous injections and trial bleeds were done via the marginal ear vein.

Collected blood was incubated for 1 h at 37°C to facilitate clotting and then held at 4°C overnight to extrude serum. The serum was removed, centrifuged to remove residual cells and stored at -20°C in 0.5 ml aliquots. Antiserum titres were tested using the long agglutination test of Vincent [52]. No precipitation reactions occurred with the pre-inoculation sera, but strong agglutinations occurred with the test antisera. Antisera agglutination titres were 1:600, 1:200, 1:400 and 1:500 for strains PPRICI3, UCT40a, UCT44b and UCT61a, respectively.

#### Antigen preparation from roots nodules

*Cyclopia maculata *seedlings were grown on nutrient-agar slants in individual sterile tubes. After three weeks of growth, the tubes were inoculated with test strains using three replicate tubes per strain and three uninoculated tubes as a negative control. The inoculated treatments included pure cultures of the four test strains and soil washes prepared from three field soils. The soils were sampled from three farms across the Western Cape: Waboomskraal near George (33° S, 22° W, CD), Kanetberg near Barrydale (33° S, 20° W, DD) and Reins Farms near Gourtismond (34° S, 21° W, BC). The three fields had no history of *Cyclopia *cultivation or the species. Nodules were harvested from the seedlings after sixteen weeks of growth. For each strain, three large nodules were harvested per replicate tube and 10 nodules per tube for each soil wash treatment. This gave a total of 9 nodules (all containing the same antigen) for each rhizobial strain and 30 nodules for each soil-wash treatment (with all 30 nodules probably containing different antigens). Antigens were extracted from the nodules by crushing individual nodules (mass ≈ 0.15 g) in 50 μl PBS and transferring 10 μl of the nodule macerate into 1 ml PBS (to give a low antigen concentration). The antigens were stored in 1.5 ml Eppendorf vials at 0°C and used within 48 h.

#### Testing the analytical sensitivity of antigen × antibody reactions

Checkerboard assays were carried out to determine the concentration effect of primary antibody (described above) and secondary antibody-conjugate (goat anti-rabbit antibody conjugated to alkaline-A-phosphatase, purchased from Sigma-Aldrich Chemical Co. Ltd.) on the sensitivity of antigen detection. The primary antibody concentration had no effect on absorbance readings, whereas a lower secondary antibody concentration of 1:4000 (diluted in 1% non-fat milk-PBS solution) significantly increased the analytical sensitivity of the test (data not shown).

Two sets of cross-reaction tests were carried out. The first used the antigens prepared from the four test strains (9 antigens per strain) and the second the soil-wash antigens (90 antigens prepared from three field soils). All possible primary antibody × antigen combinations were tested in duplicates. Wells of polysorp immunoplates (AEC-Amersham Co.) were coated with 100 μl of antigen and left at 5°C overnight. The plates were then washed three times with PBS (250 μl per well) and blocked with 200 μl 1% non-fat milk in PBS per well. After incubating at room temperature for two hours, 100 μl of the appropriate primary antibody (1:4000 diluted in 1% non-fat milk-PBS) was added to each well and the plates incubated for two hours at room temperature. After washing in PBS, 100 μl of secondary antibody was added to each well (1:4000 diluted in 1% non-fat milk-PBS) and the plates incubated at 37°C for one hour before washing (as before). Finally, a chromogenic enzyme substrate, *p*-nitrophenyl phosphate in 10% Tris-HCl buffer (Sigma-Aldrich chemical Co.), was added at the rate of 100 μl per well and the plates incubated in the dark and read when absorbance readings reached 1.0 OD_405 _for positive controls (approximately 30 min).

## Results

### Antibiotic Resistance

#### Intrinsic natural resistance of *Cyclopia *strains to low antibiotic concentrations

**Figure 1:**
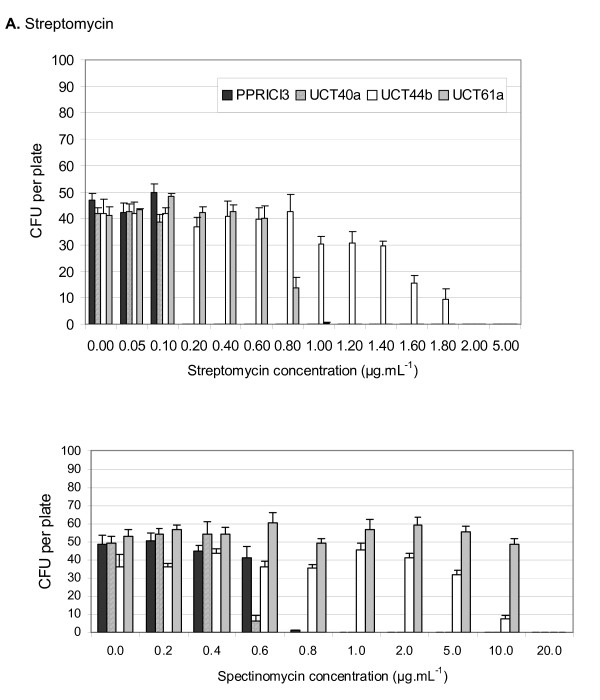
**Intrinsic natural resistance of *Cyclopia *rhizobial strains to low concentrations of streptomycin sulphate (A) and spectinomycin dihydrochloride pentahydrate (B)**. Values are mean colony-forming units (CFU) per plate (n = 3 and error bars represent standard errors).

The *Cyclopia *strains fell into two distinct groups regarding their intrinsic antibiotic resistance, with strains UCT44b and UCT61a showing greater resistance than strains UCT40a and PPRICI3 to low concentrations of both streptomycin and spectinomycin (Figure 1). Strain UCT44b was tolerant to 1.4 – 1.6 μg ml^-1 ^streptomycin and to 5.0 – 10 μg ml^-1 ^spectinomycin. Strain UCT61a showed a slightly lower tolerance to streptomycin (about 0.6 – 0.8 μg ml^-1^) but exhibited a higher tolerance of spectinomycin (about 10.0 – 20.0 μg ml^-1^). Strains UCT40a and PPRICI3, on the other hand, were highly sensitive to low concentrations of the two antibiotics, with resistance to 0.1 – 0.2 μg ml^-1 ^streptomycin and 0.4 – 0.8 μg ml^-1 ^spectinomycin.

#### Nodulation and competitive ability of antibiotically-marked versus unmarked strains

The uninoculated control plants were not nodulated and thus showed significantly lower plant dry matter yield compared to the inoculated (nodulated) seedlings (*P *< 0.01, Table 2). The nodulation and N_2_-fixing ability of the mutants of strains PPRICI3, UCT44b and UCT61a were not altered by the antibiotic marker, as there were no significant differences in plant biomass, nodule mass or nodule number between strains (*P *< 0.05, Table 2). Marked strain UCT40a_*Mkd*3 _produced no nodules, thus showing loss of symbiotic ability. Mutant strains UCT40a_*Mkd*1 _and UCT40a_*Mkd*2 _however showed no loss of their nodulation capacity compared to their parent strain (Table 2).

**Table 2:** Nitrogen-fixing ability of marked rhizobial strains.

Treatment	Total dry weight (mg)	Nodule biomass (mg)	Nodule number
Uninoculated	0.06 ± 0.04 a	0.00 ± 0.00 a	0.0 ± 0.0 a
Inoculated	0.72 ± 0.01 b	33.33 ± 0.07 b	19.6 ± 0.1 b
			
**t_(1,83)_**	2.58 **	2.60 **	3.49 **

PPRICI3 Parent	0.87 ± 0.13	18.60 ± 0.64	14.8 ± 0.5
PPRICI3*Mkd1*	0.70 ± 0.14	23.60 ± 0.78	13.2 ± 0.7
PPRICI3*Mkd2*	0.68 ± 0.10	15.40 ± 0.48	11.2 ± 0.5
PPRICI3*Mkd3*	1.26 ± 0.13	18.00 ± 0.62	12.6 ± 0.5
			
**F_(3,16)_**	**2.06 ns**	**0.51 ns**	**0.17 ns**

UCT40a Parent	2.26 ± 0.19 a	75.76 ± 1.36 a	20.0 ± 0.7 a
UCT40a*Mkd1*	1.83 ± 0.23 a	74.70 ± 1.38 a	24.3 ± 0.7 a
UCT40a*Mkd2*	2.13 ± 0.20 a	81.94 ± 1.20 a	31.6 ± 0.7 a
UCT40a*Mkd3*	0.12 ± 0.06 b	0.00 ± 0.00 b	0.0 ± 0.0 b
			
**F_(3,16)_**	4.35 *	10.30 **	8.13 **

UCT44b Parent	0.37 ± 0.13	31.25 ± 0.43	18.0 ± 0.4
UCT44b*Mkd1*	0.90 ± 0.12	56.00 ± 0.81	33.4 ± 0.8
UCT44b*Mkd2*	0.51 ± 0.09	23.20 ± 0.47	18.4 ± 0.5
UCT44b*Mkd3*	0.66 ± 0.12	25.60 ± 0.60	18.2 ± 0.6
			
**F_(3,16)_**	**1.61 ns**	**2.22 ns**	**2.94 ns**

UCT61a Parent	0.84 ± 0.12	39.82 ± 0.93	25.4 ± 0.7
UCT61a*Mkd1*	0.54 ± 0.09	22.64 ± 0.44	16.0 ± 0.5
UCT61a*Mkd2*	0.61 ± 0.10	34.02 ± 0.73	21.6 ± 0.5
UCT61a*Mkd3*	1.07 ± 0.14	48.10 ± 1.04	32.0 ± 0.8
			
**F_(3,16)_**	**2.79 ns**	**1.63 ns**	**1.79 ns**

Values are mean ± SE (n = 5) and different letters within a column indicate significant differences. ** Denotes a significant effect at *P *< 0.01, * = *P *< 0.05 and ns = no significant effect.

The antibiotically-marked strains showed varied abilities to compete with their parent strains for nodule occupancy (Table 3). The mutants of UCT44b and UCT61a showed significantly reduced competitive abilities, while those of PPRICI3 retained their competitiveness relative to the parent strain. Marked strain UCT40a_*Mkd*2 _also retained its competitive ability, while mutant strain UCT40a_*Mkd*1 _showed increased competitive ability compared to its unmarked parent (Table 3). The marked strains also varied in their retention of the antibiotic marker after plant passage (Table 4). Mutants of strain PPRICI3 retained their resistance marker, while those of UCT40a and UCT44b showed a slight reduction in the number resistant to antibiotics. Two of the three UCT61a mutants (i.e. UCT61a_*Mkd*1 _and UCT61a_*Mkd*2_) lost their antibiotic markers after plant passage (Table 4).

**Table 3:** Competitiveness of antibiotically-marked strains compared to their unmarked parents.

Treatment	Number of isolates tested	Number able to grow on YMA + antibiotics	% nodule occupancy by marked strain	Competitive ability of marked strain
UCT40a + UCT40a*Mkd1*	40	30	75.0 *	I
UCT40a + UCT40a*Mkd2*	28	14	50.0	U

UCT44b + UCT44b*Mkd1*	18	4	22.2 *	R
UCT44b + UCT44b*Mkd2*	38	12	31.6 *	R
UCT44b + UCT44b*Mkd3*	26	10	38.5	U

UCT61a + UCT61a*Mkd1*	50	0	0.0 *	R
UCT61a + UCT61a*Mkd2*	52	0	0.0 *	R
UCT61a + UCT61a*Mkd3*	60	0	0.0 *	R

PPRICI3 + PPRICI3*Mkd1*	35	21	60.0	U
PPRICI3 + PPRICI3*Mkd2*	31	19	61.2	U
PPRICI3 + PPRICI3*Mkd3*	31	10	32.3	U

* Denotes significant deviation from the expected frequency of 50% nodule occupancy using a χ^2 ^test on pooled data, *P *< 0.05. Symbols indicate I = increased, U = unchanged and R = reduced competitive ability of the marked strain compared to its unmarked parent.

**Table 4:** Retention of the antibiotic resistance marker after plant passage.

Marked Strain	Number of isolates tested	Number able to grow on YMA + antibiotics	% retention of antibiotic resistance
UCT40a*Mkd 1*	25	23	92
UCT40a*Mkd2*	25	25	100

UCT44b*Mkd 1*	20	20	100
UCT44b*Mkd 2*	21	17	81
UCT44b*Mkd 3*	19	16	84

UCT61a*Mkd 1*	15	0	0
UCT61a*Mkd 2*	14	0	0
UCT61a*Mkd 3*	13	13	100

PPRICI3*Mkd 1*	19	19	100
PPRICI3*Mkd 2*	19	19	100
PPRICI3*Mkd3*	20	20	100

#### Indirect ELISA assays

**Figure 2:**
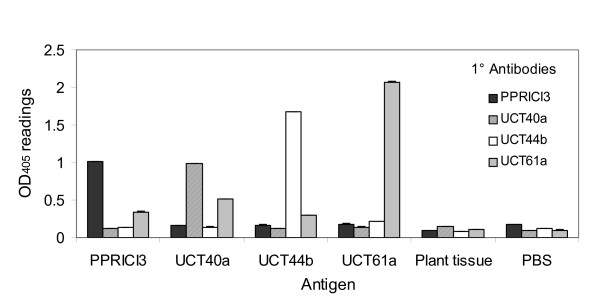
**Cross-reaction tests of indirect ELISAs involving primary antibodies assayed against 4 test antigens, with plant tissue and PBS as controls**. Nine antigens prepared for each test strain were assayed in duplicates. Error bars representing standard errors ranged from 0.001 – 0.006 OD405.

Results of the cross-reaction tests using pure antigens of PPRICI3, UCT40a, UCT44b and UCT61a (isolated from nodules of plants inoculated with these strains) are shown in Figure 2. Absorbance readings were clear and unambiguous; there were no cross-reactions, i.e. no false positive results for non-appropriate antigen × antibody combinations. In addition, non-specific adsorption using plant tissue or PBS substrate was low (≤ 0.15 OD_405_). There were some variations in the reactivity of the primary antibodies. For example, antibodies raised against strains UCT44b and UCT61a produced readings of ≥ 1.50 OD_405_, while strains PPRICI3 and UCT40a gave lower positive readings of about 1.0 OD_405_. The negative readings for all strains were ≤ 0.50 OD_405_, but were higher for strain UCT40a than the other three test strains.

Cross-reaction tests using random antigens extracted from three field soils produced less defined readings with a number of distinct cross-reactions (Table 5). The primary antibodies raised against strains UCT40a and UCT61a gave absorbance readings that were unambiguously negative (≤ 0.30 OD_405_). Optical density readings were higher (≤ 0.50 OD_405_) for the antibody raised against strain UCT44b, but all readings were distinguishable as negative. The readings for the primary antibody raised against strain PPRICI3, on the other hand, were ambiguous (≥ 0.50 OD_405_) as the antibody produced many false positive readings (≥ 1.0 A_405_). The cross-reactions were more than 50% for each of the three field soils with the primary antibody of strain PPRICI3. Antigens isolated from the soil of Rein's Farms notably produced 90% false positive readings with the primary antibody raised against strain PPRICI3 in the indirect ELISA test (Table 5).

**Table 5:** Cross-reaction tests of indirect ELISAs involving primary antibodies assayed against random antigens extracted from 3 different field soils.

Antigen (field soil site)	1° antibody			
	**PPRICI3**	**UCT40a**	**UCT44b**	**UCT61a**
Waboomskraal	60	0	0	0
Rein's Farms	90	0	0	0
Kanetberg	55	0	3	0

Data are % antigens tested positive (≥ 1.0 OD_405_), n = 30, assayed in duplicates.

## Discussion

### Suitability of intrinsic antibiotic resistance for identification of *Cyclopia *rhizobia

The four *Cyclopia *strains fell into two distinct pairs with regard to their intrinsic natural resistance to the antibiotics streptomycin and spectinomycin. In the 0.0 – 0.1 μg ml^-1 ^range, all four strains were resistant to streptomycin and could therefore not be distinguished by this technique. Over 0.2 μg ml^-1^, UCT40a and PPRICI3 were sensitive and did not grow, while UCT44b and UCT61a were resistant and could therefore be distinguished from the other two but not between themselves. However, from 1.2 – 1.8 μg ml^-1 ^streptomycin, only strain UCT44b could grow and this strain could therefore be detected in a mixture with the other three strains. Test strain resistance to spectinomycin was similar in pattern to streptomycin, in that, all strains were resistant to the 0.0 – 0.6 μg ml^-1 ^range, and were therefore not identifiable among them. However, between 1.0 and 10.0 μg ml^-1 ^spectinomycin, only strains UCT44b and UCT61a could grow in the medium and could therefore be distinguished from any one of the other two in a mixture, but again not between themselves. Based on the distinct groupings obtained in this study, in hindsight, it would have been interesting to screen a broader population of isolates for their intrinsic antibiotic resistance.

It is interesting to note that the strains used could also be grouped with respect to colony characteristics such as colony morphology. Strains UCT40a and PPRICI3, which showed low resistance, both form small, discrete, opaque colonies with little exopolysaccharide gum production. Evidence from molecular analysis show that these two strains are in fact the same species [57]. Strains UCT44b and UCT61a, on the other hand, were found to be genetically different from each other and from strains PPRICI3 and UCT40a [57]. They form fast-growing colonies with large quantities of translucent exopolysaccharide gum. Our data on antibiotic resistance and colony morphology of the four test strains are consistent with the findings of other studies, which show that fast-growing "wet" colonies have higher antibiotic resistance than "dry" colonies [58,59].

### Antibiotic markers as a tool for the detection of Cyclopia rhizobia

Analysis of root nodules for strain occupancy in the competition experiments conducted in Leonard jars revealed significant differences in the symbiotic ability and competitiveness of the antibiotic mutants relative to their unmarked parents. Marked strains from the intrinsically low resistance group (except strain UCT40a_*Mkd*3_) performed well, retaining their symbiotic ability, competitive capacity, and their antibiotic-resistance marker tags. Strain UCT40a_*Mkd*1 _even showed increased competitive ability compared to its parent strain. Marked strains of UCT44b and UCT61a, on the other hand, exhibited reduced competitive ability relative to their parent strains. This reduction in competitive ability was distinct for UCT61a_*Mkd*3_, which showed zero nodule occupancy in competition with its parent strain. Strains UCT61a_*Mkd*1 _and UCT61a_*Mkd*2 _also lost their competitive ability, but this was most likely a reflection of the strains being unidentifiable through losing their antibiotic marker tag. Strain UCT44b_*Mkd*1 _also showed some loss of its antibiotic resistance marker. The loss of symbiotic ability in strains with antibiotic tagging could suggest loss of their symbiotic plasmids. However because little is known about the rhizobia from native South African legumes, we also do not know anything about their plasmids and plasmid localization of symbiotic genes in these *Cyclopia *rhizobia. Whatever the case, this suggests genetic instability in the rhizobial strains isolated from *Cyclopia *species.

Only marked strains of PPRICI3 could be confidently used in competition studies in the glasshouse, as they retained their symbiotic trait, their antibiotic markers and showed unchanged competitive abilities. The antibiotic markers did not therefore allow for a full comparative study across the four test strains. The method is also dubious for field studies as strains from the high resistance group may exist in the field environment, and thus prove intrinsically resistant to the marker level used for low-level fingerprinting (Table 1). As all four strains were isolated from the same region and from the same area proposed for *Cyclopia *cultivation (the fynbos in the Western Cape of South Africa), the presence of intrinsically high-resistance rhizobia in the field is probable and may present problems when identifying antibiotically-marked strains from the low resistance group in field competition experiments. In addition, concerns have been raised regarding the consequences of releasing antibiotic-resistant bacteria into field environments [60,61,49].

### Indirect ELISA technique

The indirect ELISA technique is more suitable than the antibiotic resistance methods for identifying *Cyclopia *strains in nodules in glasshouse and field studies. There were no cross-reactions between the four test strains, showing that they are antigenically different (Figure 2). All four primary antibodies reacted strongly with their appropriate homologous strain, producing absorbance readings that were easily distinguished from heterologous strains, and thus made this technique ideal for strain identification in comparative glasshouse and field competition studies.

The antibodies raised against strains UCT40a and UCT61a did not cross-react with antigens from any of the three field soils and the antibody raised against strain UCT44b provided only one ambiguous positive result (0.69 OD_405 _with an antigen derived from the Kanetberg soil), but did not cross-react with antigens from the other field sites (Table 5). The antibody raised against strain PPRICI3, on the other hand, produced many false positive results, making the indirect ELISA method unsuitable for identifying this strain in field experiments. The reason for the high level of cross-reactions with the PPRICI3 antibody remains unclear. According to the polyphasic taxonomic investigations of Kock [53], strain PPRICI3 is genetically identical to strain UCT40a. However, because the two strains produced antibodies with different specificity levels, clearly indicates they differ in their surface antigen characteristics.

## Conclusion

The antibiotic markers were found to be unsuitable for identifying *Cyclopia *rhizobia in competition experiments under both glasshouse and field conditions. In contrast, the indirect ELISA technique was very successful in identifying the four *Cyclopia *strains under glasshouse conditions, as well as identifying strains UCT40a, UCT44b and UCT61a (but not strain PPRICI3) in field studies.

## Authors' contributions

AS conducted the studies as a PhD student in FD's laboratory, and prepared the draft paper. FD conceptualized the study, supervised all aspects of the work, and critically edited the paper. All authors read and approved the final manuscript.
